# Light gradient boosting-based prediction of quality of life among oral cancer-treated patients

**DOI:** 10.1186/s12903-024-04050-x

**Published:** 2024-03-19

**Authors:** Karthikeyan Ramalingam, Pradeep Kumar Yadalam, Pratibha Ramani, Murugesan Krishna, Salah Hafedh, Almir Badnjević, Gabriele Cervino, Giuseppe Minervini

**Affiliations:** 1 Department of Oral Pathology and Microbiology, Saveetha Dental College and Hospitals, Saveetha Institute of Medical and Technical Sciences (SIMATS), Saveetha University, Chennai, India; 2 Department of Periodontics, Saveetha Dental College and Hospital, Saveetha Institute of Medical and Technical Science (SIMATS), Saveetha University, Chennai, India; 3 Department of Oral and Maxillofacial Surgery, Saveetha Dental College and Hospitals, Saveetha Institute of Medical and Technical Sciences (SIMATS), Saveetha University, Chennai, India; 4 Orthodontics Department, Faculty of Dentistry, Sana’a University, Sana’a, Yemen; 5 Verlab Research Institute for Biomedical Engineering, Medical Devices, and Artificial Intelligence, Ferhadija 27, Sarajevo, 71 000 Bosnia and Herzegovina; 6 Dental Sciences and Morphofunctional Imaging, University of Messina - Policlinico “Gaetano Martino”, Via Consolare Valeria, Messina, ME 98100 Italy; 7 Saveetha Dental College and Hospitals, Saveetha Institute of Medical and Technical Sciences (SIMATS), Saveetha University, Chennai, Tamil Nadu, India; 8 Multidisciplinary Department of Medical-Surgical and Dental Specialties, University of Campania Luigi Vanvitelli, Naples, Italy

**Keywords:** Quality of life, Oral cancer, Machine learning, EORTC, Clinical, A.I

## Abstract

**Background and introduction:**

Statisticians rank oral and lip cancer sixth in global mortality at 10.2%. Mouth opening and swallowing are challenging. Hence, most oral cancer patients only report later stages. They worry about surviving cancer and receiving therapy. Oral cancer severely affects QOL. QOL is affected by risk factors, disease site, and treatment. Using oral cancer patient questionnaires, we use light gradient Boost Tree classifiers to predict life quality.

**Methods:**

DIAS records were used for 111 oral cancer patients. The European Organisation for Research and Treatment of Cancer’s QLQ-C30 and QLQ-HN43 were used to document the findings. Anyone could enroll, regardless of gender or age. The IHEC/SDC/PhD/OPATH-1954/19/TH-001 Institutional Ethical Clearance Committee approved this work. After informed consent, patients received the EORTC QLQ-C30 and QLQ-HN43 questionnaires. Surveys were in Tamil and English. Overall, QOL ratings covered several domains. We obtained patient demographics, case history, and therapy information from our DIAS (Dental Information Archival Software). Enrolled patients were monitored for at least a year. After one year, the EORTC questionnaire was retaken, and scores were recorded. This prospective analytical exploratory study at Saveetha Dental College, Chennai, India, examined QOL at diagnosis and at least 12 months after primary therapy in patients with histopathologically diagnosed oral malignancies. We measured oral cancer patients’ quality of life using data preprocessing, feature selection, and model construction. A confusion matrix was created using light gradient boosting to measure accuracy.

**Results:**

Light gradient boosting predicted cancer patients’ quality of life with 96% accuracy and 0.20 log loss.

**Conclusion:**

Oral surgeons and oncologists can improve planning and therapy with this prediction model.

## Background and introduction

In the current global context, the most sought-after treatment modality is the one that stresses patient autonomy [1]. Even in the treatment of patients with oral cancer, there is a tendency toward individualized management that includes numerous outcomes beyond just survival and response rate. Oral and lip cancers rank sixth globally in terms of mortality, according to statistics, with a reported rate of 10.2%. Due to the difficulty in mouth opening and swallowing, the majority of oral cancer patients only report when the condition is in advanced stages. They also exhibit anxiety about surviving a cancer diagnosis and receiving future treatment [2, 3]. The European Organisation for Research and Treatment of Cancer Quality of Life Questionnaire Head and Neck Module (EORTC QLQ-HN43) is a revised and updated version of the Head and Neck Cancer Module (QLQ-HN35) [4, 5]. It is a supplementary questionnaire module with the General Quality of Life Questionnaire Core 30 (QLQ-C30). The QLQ-HN43 incorporates twelve multi-item scales to assess pain in the mouth, swallowing, problems with teeth, dry mouth, sticky saliva, problems with senses, speech, body image, social eating, sexuality, problems with the shoulder, skin problems, and fear of progression. In addition, seven single items assess problems opening the mouth, coughing, social contact, swelling in the neck, weight loss, problems with wound healing, and neurological problems. Artificial intelligence (A.I.) [6–12], which imitates human cognitive processes, is a revolutionary technology that has captured the attention of scientists worldwide [13–29].

Machine learning algorithms were developed to predict reduced health-related quality of life (HRQoL) with high accuracy in patients with benign or low-grade brain tumors, suggesting they can predict symptoms and global HRQoL decline up to 60 months post-surgery [30]. The previous study aimed to determine if machine learning (ML) algorithms could predict HRQOL improvements after stroke sensorimotor rehabilitation. Five ML algorithms were used, with random forest and k-nearest neighbors effectively predicting recovery.

Important predictors included age, gender, baseline HRQOL, wrist and hand muscle function, arm movement efficiency, and sensory function. Hecksher coined the term “quality of life” (QOL), which the U.S. National Library of Medicine accepted as a keyword in 1977. The World Health Organization defines “quality of life” as a person’s assessment of their place in life within the framework of their culture and in connection to their aspirations, norms, expectations, and worries [13, 31]. Previous studies compared the management of older and younger patients with head and neck cancer, finding older patients had more comorbidities and stage IV tumors. Treatment options were similar, with radiotherapy being more common in older patients.

The emphasis on health measurement has recently shifted away from conventional metrics like mortality and morbidity. Comprehensive treatment planning must include indications that show how disease and impairment affect daily activities and behavior, subjective health, and disability or functional status [3]. In oral healthcare, machine learning-based illness prediction utilizing clinical data parameters seems promising. Advanced algorithms like light gradient boost trees analyze and interpret massive patient data to improve forecasts, early detection, and personalized treatment strategies. Clinical factors such as patient demographics, medical history, test results, and imaging findings feed these algorithms. An algorithm learns patterns and relationships in data by training a machine learning model on a dataset with known outcomes. Light Gradient Boosting Tree (LightGBM) [32] improves gradient boosting, training efficiency, and prediction accuracy in huge datasets. The ensemble machine learning technique Random Forest aggregates many decision trees to improve forecast accuracy and handle complex datasets. Few studies have been done to predict quality of life based on questionnaires using advanced machine learning. Predicting quality of life helps identify patients at higher risk of negative impacts, enabling targeted interventions like psychological support or symptom management strategies for those needing additional assistance [33–36]. We aim to predict the quality of life using light gradient Boost Tree classifiers based on questionnaires from oral cancer patients.

## Methods

Thirty-five closed-ended questions regarding oral cancer pre- and post-treatment were formulated. The validation committee, comprised of postgraduate students and faculty from Saveetha Dental College’s Department of Oral Pathology in Chennai, examined each question. Statistical analysis was used to estimate the size with a sample size of 201, a confidence level of 95%, a margin of error of 5%, and an expected failure rate of 20%. Two hundred-one patients participated in a questionnaire study that we performed using the snowball sampling technique. Of the 201 participants, 111 offered their time to participate in the survey. Use the web data protocol to swiftly and securely retrieve data and gather responses. The European Organisation for Research and Treatment of Cancer Quality of Life Questionnaire Head and Neck (EORTC QLQ-H&N) [5, 37] is a patient-reported outcome (PRO) measure designed to assess patients’ quality of life with head and neck cancer. It is a comprehensive questionnaire that covers a wide range of domains, including physical, functional, emotional, and social well-being. The Declaration of Helsinki conducted the study, and the Ethics Committee of the Institute approved the protocol, Saveetha Dental College And Hospitals [Protocol number: IHEC/SDC/PhD/0 PATH-2212/22/001; Date: 23/08/2022]. The study protocol was developed, and all subjects gave their written informed consent for inclusion before participating.

After obtaining informed consent from the patients, the EORTC QLQ-HN43 and QLQ-C30 questionnaires were given to them. It was used both in Tamil and English. Various domains were documented, such as pain, appearance, and oral function.

(EORTC QLQ-HN43) is a revised and updated version of the Head and Neck Cancer Module (QLQ-HN35). It is a supplementary questionnaire module with the General Quality of Life Questionnaire Core 30 (QLQ-C30). The QLQ-HN43 incorporates twelve multi-item scales to assess pain in the mouth, swallowing, problems with teeth, dry mouth, sticky saliva, problems with senses, speech, body image, social eating, sexuality, problems with the shoulder, skin problems, and fear of progression. In addition, seven single items assess problems opening the mouth, coughing, social contact, swelling in the neck, weight loss, problems with wound healing, and neurological problems. EORTC QLQ-C30 has 30 questions that must be taken, along with 43 questions in QLQ-HN43, which has 73 questions. Other questionnaires, like the Oral Health Impact Profile-14 (OHIP-14), are also available but not widely used. Hence, we studied the QOL using the validated EORTC QLQ-HN43 and QLQ-C30 questionnaires.

The EORTC QLQ-H&N was developed by a multidisciplinary group of head and neck cancer experts, including patients, clinicians, and researchers. It has been extensively validated and is now widely used in clinical trials and research studies to assess the impact of head and neck cancer and its treatment on patients’ quality of life.

The questionnaire consists of 35 items, each scored on a 4-point scale ranging from “not at all” to “very much.” The items are grouped into nine subscales:

- Functional impairment.
- Pain.
- Emotional functioning.
- Social eating.
- Social contact.
- Speech problems.
- Swallowing problems.
- Sensory problems.
- Global health status/quality of life.

The EORTC QLQ-H&N is a valuable tool for assessing the quality of life of head and neck cancer patients. It can be used to track changes in quality of life over time, to identify areas of concern, and to evaluate the effectiveness of different treatment strategies. EORTC QLQ-C30 has 30 questions that have to be taken, along with 43 questions in QLQ-HN43, which has 73 questions. Other questionnaires, like the Oral Health Impact Profile-14 (OHIP-14), are also available but not widely used. Hence, we studied the QOL using the validated EORTC QLQ-HN43 and QLQ-C30 questionnaires. We have compared the overall health and QOL scores obtained with the clinical and demographic details of our patients with oral cancer.

The Saveetha Dental College’s SRB Committee in Chennai, India, provided ethical approval. The Declaration of Helsinki was followed in the gathering of data and the formulation of recommendations. Questionnaire data was collected, and data was preprocessed, outliers removed, choosing a model, training, evaluating, adjusting hyperparameters, cross-validating, and making the data robot tool interpretable by Light Gradient Boosted Trees (https://app.datarobot.com). All other models showed less accuracy.

### Light gradient boosted trees

Light Gradient Boost Trees (LightGBM) is a machine learning algorithm for large-scale datasets. It uses gradient descent optimization and iterative weight updates to minimize loss. Its unique features, including a histogram-based approach for best-split points, Leaf-wise tree growth strategy, and customized data storage layout, contribute to its exceptional speed and accuracy.

The architecture of LightGBM involves several key components:

1. **Histogram-based Learning**: LightGBM uses a histogram-based approach for tree construction, which helps reduce memory usage and speeds up the training process. Instead of using the exact values of feature points, it constructs histograms to represent the distribution of feature values.
2. **Leaf-wise Tree Growth**: Unlike traditional depth-wise tree growth, LightGBM grows trees leaf-wise. It selects the leaf node with the maximum delta loss during tree growth. This approach tends to result in a more accurate model but may lead to overfitting, so regularization techniques are applied to control it.
3. **Gradient-based One-Side Sampling (GOSS)**: LightGBM uses GOSS to perform efficient gradient-based sampling during training. This technique helps to select the instances with large gradients, focusing on the samples that contribute the most to the error.
4. **Exclusive Feature Bundling**: LightGBM supports exclusive feature bundling, which groups categorical features with common values. This can help improve the algorithm’s efficiency when dealing with categorical features.
5. **Parallel and GPU Learning**: LightGBM is designed for distributed computing and supports parallel and GPU learning. This makes it suitable for large datasets and accelerates the training process.
6. **Regularization**: LightGBM incorporates regularization techniques such as L1 and L2 to prevent overfitting during training.

## Results

The included patients were of both male and female gender (56% male, 44% female). They were in the age range of 30–70 years, with a mean age of 50. The study samples included tobacco users (82%) and non-tobacco users (18%). Smoking tobacco was identified in 32%; tobacco chewing was noted in 31% of patients; and 3% smoked and chewed tobacco.

The performance of Light Gradient Boosting (LightGBM) can be evaluated using various metrics, such as accuracy, recall, F1 score, and area under the receiver operating characteristic curve (AUC-ROC). It is often faster and more memory-efficient than other boosting algorithms, such as XGBoost and AdaBoost. LightGBM can handle categorical features directly without requiring one-hot encoding, which can save time and memory. Moreover, LightGBM provides options for controlling the trade-off between computation time and model accuracy. Parameters like the number of iterations, learning rate, number of leaves, and max depth can be adjusted to optimize performance for specific use cases.

Light gradient boosting predicted cancer patients’ quality of life with 96% accuracy and 0.20 log loss.

### AUC-ROC

The AUC-ROC is a metric used to evaluate binary classification models, indicating their performance distinguishing positive and negative instances. It ranges from 0 to 1, with higher values indicating better performance. A high AUC-ROC indicates a good balance between sensitivity and specificity and a high chance of ranking positive instances higher than negative ones.

## Discussion

Artificial intelligence (AI) is a relatively new technology with great predictive potential. With so much digital data at their disposal, AI has the amazing potential to enable meaningful decisions in choosing the best treatment for every patient. EORTC scoring is one to seven, with one (very poor) to seven (excellent). We documented the overall quality of life score at diagnosis and one year after surgical intervention for 111 patients with oral cancer. The EORTC quality of life questionnaires (QLQ-C30 and QLQ-H&N 35) were validated in India, with 200 head and neck cancer patients completing the questionnaires at two treatment points, proving their reliability and validity, similar to Previous studies translated. They validated the EORTC QLQ-H&N35 in Urdu [37], assessing its convergent and discriminant validity. The translations were comprehensible for all patients, with Cronbach alpha ranging from 0.75 to 0.98. The patient-reported content validity index scores were excellent, and weak bidirectional correlations were found with resilience, depression, and anxiety. Another study found that lower QoL scores at diagnosis and during the first year after diagnosis have a predictive value for patients with head and neck squamous cell carcinoma, independent of other factors, predicting lower overall survival [2, 3], and another study examined the correlation between three commonly used instruments for assessing the quality of life of 33 head and neck cancer patients at Mato Grosso Cancer Hospital in Brazil, revealing a positive correlation [31, 38, 39]. None of the studies did predictive analysis. So, we applied machine learning algorithms like Light Gradient Boosting Tree (LightGBM) and built trees faster using histograms, making it appropriate for huge datasets. LightGBM performs well across challenges with leaf-wise tree development and gradient-based one-side sampling. There are two fundamental differences between random forests and gradient-enhancing boost trees. Sequentially training the former corrects mistakes in the preceding trees. However, we build trees in a random forest independently. A forest can be trained with parallel but not gradient-boosting trees. Random forest trees can output in any order because they are independent [40–42]. The role of nutrition and old age in the quality of life of cancer patients is a critical aspect of cancer care. Nutrition is crucial for cancer treatment, influencing treatment outcomes, energy levels, and immune function. In older adults, age-related factors complicate the impact of cancer and treatments on quality of life. Balancing nutrition interventions with age-related considerations is essential for maintaining and improving quality of life during cancer treatment.

**Fig. 1:**
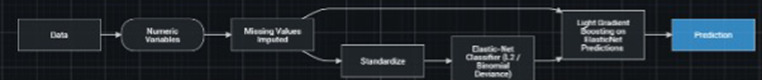
Shows a flow chart diagram of this light gradient boosting

**Fig. 2:**
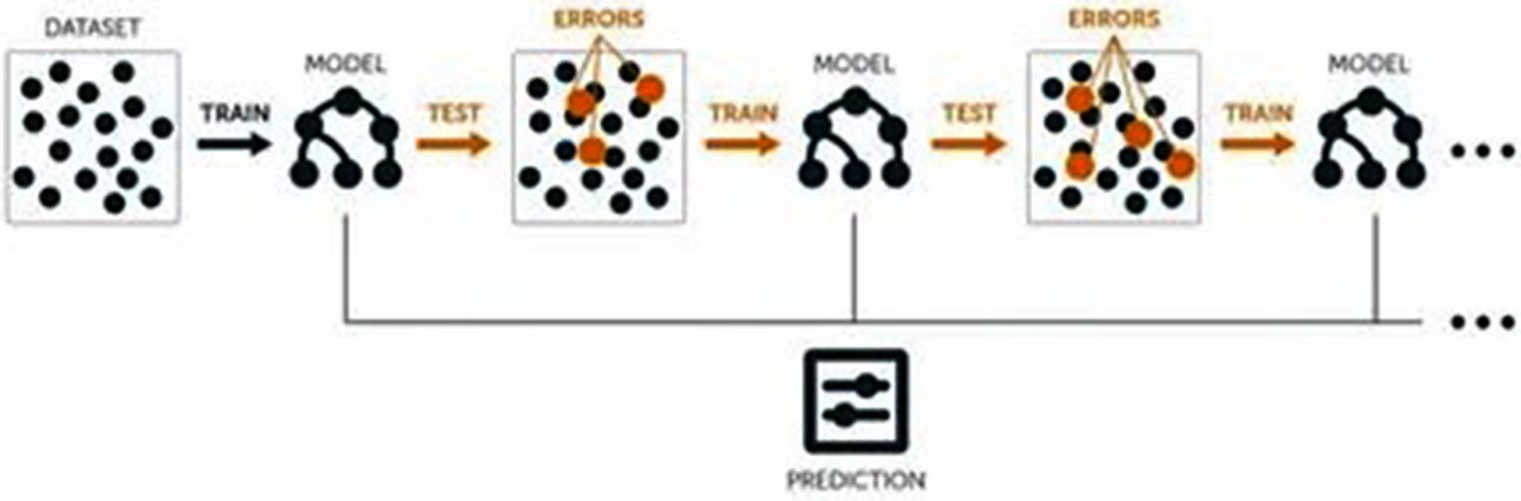
Light gradient boosting algorithm

**Fig. 3:**
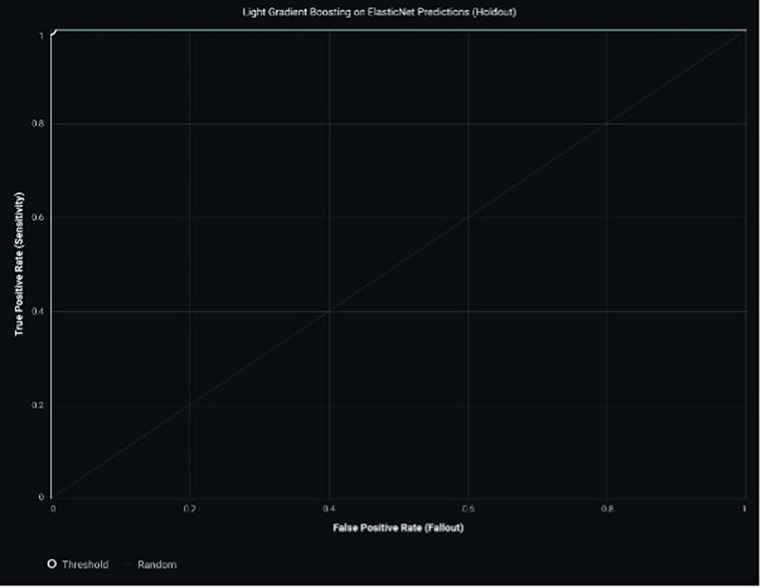
ROC curve of the predicted class

**Fig. 4:**
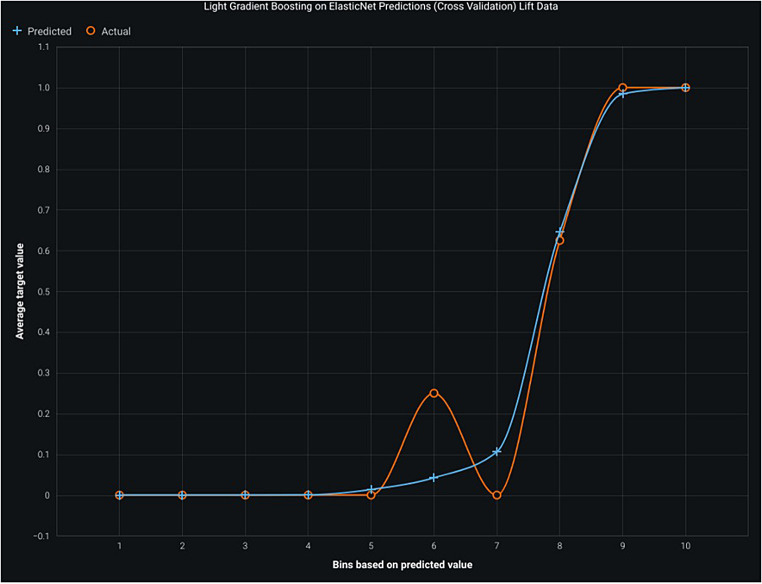
Shows lift data of elastic net predictions

However, gradient-boosting trees have a predetermined order that cannot be altered. Another study showed that the Arabic version of the MD Anderson Dysphagia Inventory was validated among 82 Saudi Arabian head and neck cancer patients [41, 43], demonstrating 100% feasibility, acceptable test-retest reliability, and concurrent validity with the EORTC Quality-of-Life Head and Neck Module [41, 44, 45]. This study concluded with pre-and post-operative results showing good improvement in quality of life and good accuracy of predictive modeling with an accuracy of 96% (Figs. 1, 2, 3 and 4, and Table 1), Another study compared QoL in patients with T1a glottic carcinoma treated with surgery or radiotherapy in U.K. specialist units [32, 40, 46–48]. Results showed similar overall QoL scores, with modest differences in certain subscales but not persisting beyond four months [49], supporting the transoral laser microsurgery recommendation. LightGBM may struggle with unbalanced datasets with many samples in one class and lack interpretability. In questionnaire-based studies, oversampling the minority class or altering class weights may be needed to make more balanced predictions. Further, large samples with better algorithms may help us achieve good accuracy for clinical applications.

**Table 1:** Shows the confusion matrix of the predicted class

Matrix: confusion matrix (percent)			
	Predicted		
Actual		n	y
	n	72.73% (TN)	0.00% (FP)
	y	0.00% (FN)	27.27% (TP)

## Conclusion

In conclusion, the development and implementation of a prediction model based on quality of life in oral cancer patients can greatly enhance the planning and therapeutic processes for oral surgeons and oncologists. This model enables a more personalized approach to care, empowering patients and optimizing resource allocation to ensure the delivery of high-quality, patient-centered care.

## Data Availability

The data will be available on reasonable request from the corresponding author.
